# Undesirables in Mesopelagic Species and Implications for Food and Feed Safety—Insights from Norwegian Fjords

**DOI:** 10.3390/foods9091162

**Published:** 2020-08-24

**Authors:** Martin Wiech, Marta Silva, Sonnich Meier, Jojo Tibon, Marc H. G. Berntssen, Arne Duinker, Monica Sanden

**Affiliations:** Institute of Marine Research, Nordnes, P.O. Box 1870, NO-5817 Bergen, Norway; Marta.Silva@hi.no (M.S.); sonnich@hi.no (S.M.); jojo.tibon@hi.no (J.T.); Marc.Berntssen@hi.no (M.H.G.B.); Arne.Duinker@hi.no (A.D.); Monica.Sanden@hi.no (M.S.)

**Keywords:** mesopelagic, contaminants, undesirables, trace elements, arsenic, fluoride, organic pollutants, wax esters, *Benthosema glaciale*, *Maurolicus muelleri*

## Abstract

The increase in the global population demands more biomass from the ocean as future food and feed, and the mesopelagic species might contribute significantly. In the present study, we evaluated the food and feed safety of six of the most abundant mesopelagic species in Norwegian fjords. Trace elements (i.e., arsenic, cadmium, mercury, and lead), organic pollutants (i.e., dioxins, furans, dioxin-like polychlorinated biphenyls, and polybrominated flame-retardants), and potentially problematic lipid compounds (i.e., wax esters and erucic acid) were analyzed and compared to existing food and feed maximum levels and intake recommendations. Furthermore, contaminant loads in processed mesopelagic biomass (protein, oil, and fish meal) was estimated using worst-case scenarios to identify possible food and feed safety issues. While most undesirables were low considering European food legislation, we identified a few potential food safety issues regarding high levels of fluoride in Northern krill, wax esters in glacier lanternfish, and long-chain monounsaturated fatty acids in silvery lightfish. Our estimates in processed biomass indicated high levels of undesirable trace elements in the protein fraction, frequently exceeding the maximum levels for feed ingredients. However, in fish meal, almost no exceedances were seen. In the oil fraction, dioxins and furans were above the maximum levels, given for food and feed ingredients. The present study is crucial to enable an evaluation of the value of these species; however, more data is needed before proceeding with large-scale harvesting of mesopelagic biomass.

## 1. Introduction

The global population is predicted to increase to 9.6 billion by 2050, demanding global food production to grow by 60% (WHO. Zero hunger—Hunger facts, http://www.fao.org/zhc/hunger-facts/en/). Seafood, being highly nutritious, has great potential to contribute to food security [1]. Marine resources can either be consumed directly by humans, processed before human consumption, or used as feed ingredients for aquaculture. Marine oils for human consumption and fish protein powder can be applied for food fortification and the production of value-added/functional foods. The continuous demand for alternative protein and oil sources for aquaculture, due to its short supply, high prices, and competition with human food, makes the exploration of new marine resources highly relevant.

However, the overfishing of commercially exploited fish stocks is still a growing issue [2]. It has been suggested that one way to reduce fishing pressure on already overfished stocks would be to harvest from so far unexploited resources, preferentially from lower trophic levels, such as organisms from the mesopelagic zone [3].

Mesopelagic organisms are a prominent, almost totally unexploited resource. They are globally distributed and inhabit the ocean waters between 200 and 1000 m of depth. They are assumed to be one of the world’s largest unexploited resources, with stock estimates of mesopelagic fish ranging from approximately one to ten billion metric tons [4,5,6]. In addition to fish species, the mesopelagic community also contains potentially exploitable species of crustaceans, jellyfish, and cephalopods.

Due to their extended deep-water zones, Norwegian fjords are a promising habitat for mesopelagic species, and it has been shown that considerable nutrient-dense biomass can be found there. The species variety is rather low, and the biomass consists of mainly six species: two species of mesopelagic fish, the glacier lanternfish (*Benthosema glaciale*), and the silvery lightfish (*Maurolicus muelleri*); the decapod *Eusergestes arcticus*; the decapod genus *Pasiphaea*; the euphausiid Northern krill (*Meganyctiphanes norvegica*); the scyphozoan helmet jellyfish (*Periphylla periphylla*) [7]. It has been shown that these species have the potential to contribute to global food and feed security being nutrient-rich with high levels of vitamin A_1_, calcium, selenium, iodine, eicosapentaenoic acid, docosahexaenoic acid, and cetoleic acid, especially in fish [7].

However, more knowledge on the content of undesirables is needed to assess the suitability as food and feed ingredient, especially since some challenges have already been identified, such as high values of cadmium in some fish species [7,8,9] and fluoride in Northern krill [10,11] and high amounts of wax esters in *B. glaciale* [9,12].

The mesopelagic fisheries are so far in an experimental stage, and before exploiting the mesopelagic biomass as food or feed ingredient, it should be studied how this will impact the services of the mesopelagic organisms provide for the ocean and the climate with its function as carbon pump [13]. Therefore, the final development of the fisheries and final products and applications of the catch are not yet known. The species composition of mesopelagic catches can vary significantly, and at this point in time, we do not know yet if sustainable catches are possible, and if so, what the catches will be used for, and how the processing may influence the nutrient and contaminant composition. However, the first steps are needed to identify possible drawbacks regarding food and feed safety. Depending on the final product, different regulations might apply. In the European context, regulations setting maximum levels (MLs) for different types of contaminants are in place, both for food [14] and feed ingredients [15].

In the present study, we investigated the levels of the trace elements arsenic (As), cadmium (Cd), mercury (Hg), and lead (Pb) in the most abundant mesopelagic species in western Norwegian fjords, whose genus or family are found widespread and highly abundant in mesopelagic ecosystems all around the globe [4,16,17,18]. Samples were also analyzed for organic legacy pollutants, including dioxins and dioxin-like polychlorinated biphenyls (PCBs), non-dioxin-like PCBs (PCB6), and the content of polybrominated diphenyl ethers (PBDE_7_). Finally, the content of the indigestible wax esters and the fatty acid—erucic acid—was evaluated. Where applicable, the measured concentrations were compared to legal MLs. Furthermore, the contents of the analyzed contaminants in the protein concentrate fraction and marine oil fraction were roughly estimated using simple assumptions to enable the identification of possible food and feed risks.

## 2. Materials and Methods

### 2.1. Biological Material

The two fish species of glacier lanternfish, *Benthosema glaciale*, Pearlside, *Maurolicus muelleri*; the decapod species *Eusergestes arcticus*; the decapod genus *Pasiphaea*, including the three species *Pasiphaea multidentata, Pasiphaea sivado*, and *Pasiphaea tarda*; Northern krill, *Meganyctiphanes norvegica;* the jellyfish species helmet jellyfish, *Periphylla periphylla* were sampled in three different fjords of the Norwegian west coast—Osterfjorden, Bjørnafjorden, and Boknafjorden. Specimens were caught in a mesopelagic trawl between 5 and 9 December 2018, onboard the research vessel “Johan Hjort”. Fish and crustacean species were identified, and the standard length was measured for a representative number of animals from the catch (*n* ≥ 27). For each species/genus from each location, a minimum one pooled sample was prepared. For the *B. glaciale*, *M. muelleri*, and *M. norvegica* samples from Osterfjorden, different size classes were sampled, and for *B. glaciale*, also different sexes were determined. *P. periphylla* was only sampled from Osterfjorden (*n* = 12) and Bjørnafjorden (*n* = 10), and total wet weight (w.w.) was used as size measurement. For the pooled sample, the jellyfish individuals were quartered. Samples were homogenized as soon as possible after the catch and distributed into different tubes for analysis. All samples were stored frozen at −20 °C until analysis, while samples for lipid analysis were stored frozen at −20 °C until 17 December 2018, and at −80 °C until analysis. A detailed overview of the samples (number of composite samples, specimens per composite samples, and the average length/weight) and images of the species are given in Alvheim et al. [7].

### 2.2. Chemical Analysis

#### 2.2.1. Trace Elements

Pooled samples of the fish and crustacean species were homogenized and subsequently freeze-dried. Moisture content was determined by comparing the weight of the sample before and after freeze-drying. The freeze-dried sample material was homogenized before performing the analysis of trace elements. This determination was performed using inductively-coupled plasma mass spectrometry (ICP-MS), as described by Julshamn et al. [19]. The method is accredited by the Norwegian Accreditation Authority, according to NS-EN 17025. The accuracy of this method is assessed by using certified reference materials (i.e., lobster hepatopancreas (TORT-3; National Research Council Canada, Ottawa, ON, Canada) and oyster tissue (SMR1566b; National Institute of Standards and Technology, Gaithersburg, MD, USA)). In brief, approximately 0.2 g of sample material was digested using 2.0 mL of nitric acid (69% *w*/*w*) in an ultra wave digestion system (UltraWAVE, Milestone, Sorisole, Italy). The tubes were capped and placed in the ultra wave system in a container with 130 mL Milli-Q^®^ (EMD Millipore Corporation, Billerica, MA, USA) water and 5 mL H_2_O_2_. The digested samples were diluted to 25 mL with Milli-Q^®^ water. The tuning of the ICP-MS was performed following the manufacturer’s instructions. A tuning solution (1 ppb tuning solution B, Thermo Fisher, in 2% HNO_3_ and 0.5% HCl) was used prior to analyses. The concentrations of As, Cd, Hg, and Pb were determined by ICP-MS (iCapQ ICP-MS, Thermo Scientific, Waltham, MA, USA) equipped with an autosampler (FAST SC-4Q DX, Elemental Scientific, Omaha, NE, USA). Data were collected and processed using the Qtegra ICP-MS software (version 2.10, 2018, Thermo Scientific, Waltham, MA, USA). The dry weight-based limit of quantification (LOQd.w.) was set to 0.005 mg/kg d.w. with a standard sample size (0.2 g). The wet weight-based LOQ for each individual sample (LOQw.w.) was determined as: LOQw.w. = LOQd.w. × % dry matter_sample_/100.

#### 2.2.2. Inorganic Arsenic

The inorganic arsenic (iAs) concentration was performed, as previously described [20,21], based on an European Committee for Standardization method (NS-EN 16802:2016, European Committee for Standardization). Briefly, after freeze-drying samples, they were ground until a homogenous material was obtained. Approximately, 0.2 g of sample was weighed into a 13 mL propylene centrifuge tube (Sarstedt, Nümbrecht, Germany), and the 10 mL of extraction solution (0.1 M HNO_3_ (trace select, ≥69.0% *w*/*w*) in 3% (*v*/*v*) H_2_O_2_ (Emsure^®^ (Merck, Darmstadt, Germany) ACS, ISO, 30% *w*/*w*)) was added. The samples were placed in a water bath for 60 min at 90 °C, 100 rpm, and subsequently cooled down to room temperature and centrifuged during 10 min at 3800 rpm (Eppendorf^®^ Centrifuge 5702, Hamburg, Germany). Prior to analysis, the soluble fraction was collected with a 5 mL disposable needle syringe and filtered through a disposable syringe filter (0.45 µm, Sartorius, Göttingen, Germany) into 1 mL polypropylene HPLC vials. During the extraction procedure, arsenite [As(III)] was oxidized to arsenate [As(V)], and the iAs concentration was determined as the sum of As(III) and As(V). This determination was done by using an external calibration curve of As(V) (Spectrascan TeknoLab, Oppegaard, Norway) and using peak areas for quantification. The LOQ of this method was 0.01 mg·kg^−1^ d.w. Certified reference material of rice (ERM-BC211; Institute for Reference Materials and Measurements, IRMM, Geel, Belgium) was used to assess the accuracy of the method. The iAs concentration was determined using an HPLC-ICP-MS (1260 HPLC, 7900ICP-MS, Agilent Technologies, Wilmington, DE, USA) and anion-exchange column (IonPac AS7, 2 × 250 mm; Dionex, Sunnyvale, CA, USA) with respective guard column (IonPac AG7, 2 × 50 mm; Dionex, Sunnyvale, CA, USA). The mobile phase solution was prepared by dissolving an appropriate amount of (NH_4_)_2_CO_3_ to reach the desired ionic strength (50 mM) in an aqueous 3% (*v*/*v*) MeOH solution (MeOH, LiChrosolv^®^, HPLC grade), followed by adjustment of pH to 10.3 with NH_3_ (25% *v*/*v*). The instrument was tuned according to the manufacturer’s instructions.

#### 2.2.3. Fluoride

Total fluoride was analyzed according to Malde et al. [22]. Briefly, the fluorine content in 0.25 or 0.50 g sample material was determined by using a selective ion electrode (Orion 94–09, Thermo Orion ionpuls fluorine electrode, Beverly, MA, USA) after dry ashing in a muffle furnace (CSF 1100, Carbolite Furnaces, Bamford, Sheffield, England) at 550 °C with sodium hydroxide as an ashing aid, in order to aid the fluoride extraction as well as avoiding loss of fluoride during the ashing process. The dry-ashed samples were dissolved in distilled water (10–15 mL) and neutralized with hydrochloric acid to a pH of 7.2–7.5, in order to avoid hydroxide fluoride interference during determination. Aliquots of 5 mL were pH adjusted to pH 5.2–5.4 with 0.5 mL total ionic strength adjustment buffer III solution, which is the optimal pH-range for fluoride determination. Reagent blanks for blank determination and standard solutions (0.100, 1.000, and 10.000 mg F/L) were used for background and concentration determination. The precision of the method was assessed with certified reference material (i.e., oyster tissue, 1566a, NIST, Gaithersburg, MD, USA).

#### 2.2.4. Crude Fat

The crude fat content was determined gravimetrically in wet homogenates using 30% isopropanol in ethyl acetate. The solution was filtered, the solvent evaporated, and the fat residue weighed. This method is accredited in accordance with ISO-EN 17025 and registered as a Norwegian Standard, NS 9402 [23].

#### 2.2.5. Determination of Dioxins, Furans, Polychlorinated Biphenyls, and Polybrominated Flame-Retardants

The concentrations of dioxins and furans (PCDD/Fs) and non-ortho PCBs, mono-ortho PCBs, and PBDE were determined by using high-resolution gas chromatography/high-resolution mass spectrometry (HRGC/HRMS), according to Berntssen et al. [24,25]. Briefly, sample material was solvent extracted by pressure (80:20 dichloromethane:hexane for PPPBDE, hexane for all other substances (*v*/*v*)) with a Dionex ASE 300 solvent extractor (Dionex Sunnyvale, CA, USA). Acid-impregnated silica was added to the extraction cell for the on-line cleanup of NDL-PCBs and PBDEs. In an external clean-up procedure, co-extracted fat was removed by adding concentrated sulfuric acid to the extract. Prior to extraction, the following surrogate internal standards were added (^13^C-labeled EDF-4147, 4097, 5999, 6999, 7999, 8999, 9999-3-4, 9999 for PCDD/F, PBDE 139 EO-5100 for PBDEs, EC-4935, 4979, 4937, 4976-3, 4976 for dioxin-like -PCBs, and PCB-53 for non-dioxin-like -PCBs (Cambridge Isotope Laboratories, Andover, MA, USA)). For PCDD/F and DL-PCBs, extracts were purified using H_2_SO_4_ on silica, multilayered silica, basic alumina, and carbon columns, respectively (FMS, Waltham, MA, USA, for solvent conditions see [26]). Following this, the samples were concentrated by pressurized evaporation (Turbovap II™ Zymark, Hopkinton, MA, USA). A mixture of ^13^C-labeled performance standards (EDF 5999 for PCDD/F and EC-4979 for DL-PCBs, Cambridge Isotope Laboratories, Andover, MA, USA) was added prior to PCDD/F and DL-PCBs determination. High-resolution gas chromatography/high-resolution mass spectrometry (HRGC/HRMS, MAT 95XL Thermo Finnigan, Bremen, Germany), equipped with a fused silica capillary column (30 m × 0.25 mm i.d. and 0.25 μm film thickness, RTX-5SILMS, Restek, Bellefonte, PA, USA), was used for analyses. According to the United States Environmental Protection Agency (USEPA) 1613 method [27], the quantification was performed according to the internal standard isotope dilution method using congener-specific relative response factors (RRFs) determined from three-point calibration standard runs (CS1–CS3, Cambridge Isotope Laboratories, Andover, MA, USA). Recovery values (%) were between 78 and 110%, and these values were calculated according to the USEPA methods [27], and PCCD/F and DL-PCB values are expressed as pg upper bound WHO-TEQ g^−1^ w.w. using the WHO-TEFs from 1998 [14]. The PCCD/F and DL-PCB under the limit of quantification (LOQ) are expressed as LOQ (upper bound). The LOQ for the other persistent organic pollutantss is given as <LOQ. Determination of NDL-PCBs was performed by GC-MS (TRACE GC Ultra™/DSQ™ Single Quadrupole GC/MS, Thermo Finnigan, Bremen, Germany) in negative chemical ionization SIM mode. The GC was equipped with a fused silica capillary column (30 m × 25 mm i.d. 25 μm film thickness HP-5MS Column, Agilent J&W, Santa Clara, CA, USA). The internal standard (IS) method was used for quantification, using congener-specific RRFs from a three-point linear external standard curve relative to the internal surrogate standard. Recovery for all congeners was validated by spiking each sample matrix with internal standards at three levels (recovery was 85–110% for NDL-PCBs). For OCPs, the extracts were purified on three sequenced solid-phase extraction (SPE) columns (Chem Elut™, BondElut^®^ C18, and BondElut^®^ Florisil columns, respectively, Varian Inc., Palo Alto, CA, USA, for solvent conditions see [26]) in an automated column system (ASPEC™ XL4, Gilson, Middleton, WI, USA). The PBDE extracts were analyzed by GC-MS (TRACE GC Ultra™/DSQ™ Single Quadrupole GC/MS, Thermo Finnigan, Bremen, Germany) equipped with an RTX-5MS capillary column (30 m × 0.25 mm i.d. 25 μm film thickness, Restek, Bellefonte, PA, USA). The recovery for PBDE and HBCD was between 81% and 118%, and quantification and recovery validation were performed, as described for the PCBs. All samples were run with one procedural blank and one in-house performance evaluation standard (homogenized salmon fillet) in batches of twelve, with a duplicate of the last sample. The LOQ was determined for each determination by using nine times the noise level (three times the limit of detection (LOD)). The LOD was statistically estimated as the analyte concentration, giving a peak signal of three times the background noise from an internal-surrogate standard-spiked procedural blank. The proficiency test, quantification quality, and assurance procedures were as validated by inter-laboratory tests (details are given by Berntssen et al. [26]). The trueness of the method was established by participating in proficiency tests of calibration material and spiked sample material (i.e., satisfactory trueness was set to −2.0 ≤ *z*-score ≤ 2.0 and repeatability as relative standard deviation RSD (%) of 10 % and better).

#### 2.2.6. Wax Esters and Erucic Acid

Wax esters and erucic acid were analyzed by gas chromatography (HP-7890A Agilent, Santa Clara, CA, USA) coupled with a flame ionization detector (GC-FID), as described in Meier et al. [28], with the nonadecanoic acid (19:0) as an internal standard. For this, anhydrous methanol containing 2 N HCl was used as a methylation agent. The fatty acids methyl esters (FAME) were extracted using 2 × 2 mL hexane. Several of the samples contained wax esters, and nonadecanol (19:0 alk) was added in the hexane extracts as internal standard, and the FAME and fatty alcohols (FAOH) were separated using solid-phase column (500 mg aminopropyl-SPE, Supelco, Bellefonte, PA, USA. The FAME fraction was eluted with 3 mL hexane + 2 mL hexane:ethyl acetate 9:1 *v*/*v*), and the FAOHs were eluted with 4 mL chloroform. To obtain a suitable chromatographic response, the extracted hexane was diluted or concentrated so that the most abundant FAME/FAOH in the mixture was approximated 150 ng/µL. One µL was injected splitless with an injection temperature of 280 °C. A 25 m × 0.25 mm fused silica capillary, coated with polyethylene-glycol of 0.25 μm film thickness, CP-Wax 52 CB (Varian-Chrompack, Middelburg, The Netherlands) was used. Helium was used as the mobile phase at 1 mL/min for 45 min and then increased to 3 mL/min for 30 min. The temperature of the flame ionization detector was set at 300 °C. The oven temperature was programmed to hold at 90 °C for 2 min, then from 90 to 165 °C at 30 °C/min and then to 240 °C at 2.5 °C/min and held there for 35 min. Fifty-nine FAME peaks and fifteen fatty alcohols peaks were selected in the chromatograms and identified by comparing retention times with a FAME standard (GLC-463 from Nu-Chek Prep. Elysian, MN, USA) and fatty alcohol standard (GLC-33-36A from Nu-Chek Prep. Elysian, MN, USA), and retention index maps and mass spectral libraries (http://www.chrombox.org/home/www.chrombox.org/index.html) were performed under the same chromatographic conditions as the GC-FID [29]. Chromatographic peak areas were corrected by empirical response factors calculated from the areas of the GLC-463 mixture. The chromatograms were integrated using the EZChrom Elite software (Agilent Technologies, Santa Clara, CA, USA).

#### 2.2.7. Estimation of Contaminant Levels in Processed Mesopelagic Biomass

To estimate contaminant levels, despite lacking specific knowledge on how the contaminants will be distributed in the oil and meal fraction after processing, assumptions were made, resulting in worst-case scenarios:

The total amount of As, iAs, F, Hg, Cd, and Pb would end up in the processed pure protein fraction and in fish meal, respectively. Fish meal was defined as total biomass adjusted to the fat content of 10% (crude fat).

The processing of fish oil was equally efficient as the here applied method for the estimation of the crude fat content. The total amount of here measured persistent organic pollutants (POPs), erucic acid, and wax esters followed the oil fraction.

The concentrations of trace elements in processed pure protein $C_{Trace\;elements}^{Protein}$ with a dry matter content of 88%, as described in the EU directive 2002/32/EC [15], was estimated as:

(1) $$C_{Trace\;elements}^{Protein}=\frac{C_{Trace\;elements}^{Meso}}{C_{Protein}^{Meso}}\;\;\;\;\times dm_{Meal}\;\;\;\;\;\;\;$$

with $\;C_{Trace\;elements}^{Meso}$ being the dry weight-based concentration of trace elements in the whole mesopelagic organism, and $dm_{Meal}$ being the dry matter content in the meal, set to 0.88 g/g.

The concentrations of the here measured POPs in fish oil $C_{POPs}^{Oil}$ was estimated as:

(2) $$C_{POPS}^{Oil}=\frac{C_{POPs}^{Meso}}{C_{Total\;fat}^{Fish}}$$

with $C_{POPs}^{Meso}\;$ being the dry weight-based concentration of POPs in the whole mesopelagic organism, and $C_{Total\;fat}^{Meso}$ being the dry weight-based fat content in the whole mesopelagic organism.

The concentration of trace elements in fish meal $C_{Trace\;elements}^{Meal}$ adjusted to content of 0.88 g/g dry matter and fat content $fat_{Meal}\;$ of 0.1 g/g was estimated as:

(3) $$C_{Trace\;elements}^{Meal}=\frac{C_{Trace\;elements}^{Meso}}{{\left(1-C_{Total\;fat}^{Meso}\right)}_{}}\;\;\;\;\times dm_{Meal}\;\times \;\;(1-fat_{Meal})$$

The concentration of POPs in the fish meal $C_{POPs}^{Meal}$ was estimated as:

(4) $$C_{POPs}^{Meal}=\;C_{POPS}^{Oil}\;\times fat_{Meal}$$

The calculations for erucic acid and wax esters were done in accordance with POPs, following formulas (2) and (4).

## 3. Results and Discussion

### 3.1. Trace Elements

The concentrations of As, iAs, Cd, Hg, Pb, F in the different species are shown in Table 1 based on dry weight (d.w.) and wet weight (w.w.).

For comparison, the literature values on the investigated species are given in Table 2.

#### 3.1.1. Arsenic and Inorganic Arsenic

The viability of mesopelagic species as an alternative food or feed source largely depends on compliance with existing legislation. In many fish and shellfish, the As concentrations can exceed the concentrations found in most terrestrial foods [45]. Consequently, seafood has been reported as one of the major sources of As in humans. However, there is no EU ML for As in seafood or marine oils intended for human consumption. In 2011, the Joint FAO/WHO Expert Committee on Food Additives (JECFA) withdrew the provisional tolerable weekly intake (PTWI) for iAs in 2011 since it was no longer considered to be protective [46].

In terms of total As, most species had low average concentrations, ranging from 0.79 to 9.5 mg/kg w.w. The observed level for *B. glaciale* of 13 ± 4 mg/kg d.w. was comparable to the total As content reported by Fowler [31], and for another species of lantern fish—*Benthosema pterotum—*of 13.8 mg/kg d.w. [8]. The result for *M. muelleri* also corresponded to the total As found in the same species in another Norwegian fjord [9]. Higher concentrations were obtained for the northern krill *M. norvegica* and the shrimp *Pasiphaea* spp. at 28 and 22 mg/kg w.w., respectively. For both fish species, a high variation was observed in individual total As concentration, with few cases exceeding the ML of 25 mg/kg for fish-based feed ingredients Previous studies reported even higher concentrations in offshore samples of *M. norvegica*, more than twice the average concentration found in the present study [31,33]. The high total As concentrations found were comparable with a previous study on mesopelagic organisms, where crustaceans, such as krill and shrimp, were found to contain elevated concentrations of total As [47]. In the studied mesopelagic species, the toxic inorganic form only existed as a small portion of the total As (<2%). Fish species—*B. glaciale* and *M. muelleri—*and the shrimp *E. arcticus* had concentrations below LOQ. Quantifiable levels were observed in other species (*M. norvegica, Pasiphaea* spp., *P. periphylla*), with the highest value found in *M. norvegica* at 0.16 mg/kg w.w. The measured iAs concentrations found were well below 2 mg/kg, which could be required by competent authorities for fish meal (EU Directive 2002/32 and amendments [15]).

Arsenic occurs in different chemical forms, and they can be found in varying concentrations in fish and other marine organisms. It is well established that As toxicity is dependent on its chemical form. In marine organisms, the toxic iAs is usually present as less than 1% of the total As [20]. Thus, As exists mostly as organic species, with the relatively non-toxic form arsenobetaine being the predominant chemical species in most marine organisms, including fish, bivalves, and crustaceans [48,49]. The As speciation data obtained for the certified reference material MURST-ISS-A2 (Antarctic krill) showed that arsenobetaine, dimethylarsinate, trimethylarsoniopropionate, and oxo-arsenosugars were the major arsenical compounds found in the Antarctic krill sample [50]. Arsenobetaine concentration found corresponded to about 45% of the extractable As. Dimethylarsinate and trimethylarsoniopropionate were present as a minor but significant fraction (approximately 5% and 10% of the total extractable As, respectively), as well as oxo-arsenosugars (approximately 20% of the total extractable As) [50]. While there are no current MLs for organic As in food and feed, potential toxic effects have been reported for methylated species and arsenolipids [51]. This highlights the need for As speciation data, especially for mesopelagic species, which so far have not been investigated extensively. Further studies devoted to As speciation will provide the basis for proper risk assessment of mesopelagic species as a food or feed resource.

#### 3.1.2. Cadmium

Rather high Cd concentrations were found in the here analyzed fish species, approaching but not exceeding the maximum levels in the food of 0.05 mg/kg w.w., given for fish intended to be consumed whole [14]. Considering the size of the here investigated species, consumption of whole fish was likely. Small fish consumed whole, including the head, and viscera are part of multiple food cultures [52,53], and the here investigated species have been shown to be nutrient-dense [7]. The concentrations of Cd were most likely so high, as whole individuals were analyzed. In fish, most Cd is located in the kidney and liver, and crustaceans accumulate Cd in the hepatopancreas [54,55]. Therefore, the Cd concentration measured in whole individuals would generally be higher than in muscle samples.

Interestingly, in comparison to the here measured concentrations (0.022 ± 0.014 mg/kg w.w.), higher levels were seen in *B. glaciale* caught offshore in the Northern Norwegian Sea (0.09 mg/kg w.w.) [9] and in the North Atlantic (0.07 mg/kg w.w.) by Grimaldo et al. [30]. The concentrations of Cd reported in *B. glaciale* from the Mediterranean Sea were much higher with a large variation (0.19 ± 0.08–0.71 ± 0.15 mg/kg d.w.) [31]. For *M. muelleri*, the findings suggested a similar pattern with much higher concentrations offshore in the Atlantic [30] compared to the concentrations found in the fjords in the present study. Closer investigations are needed to test if the here investigated mesopelagic fish species, in general, contain higher levels of Cd offshore. However, the distribution pattern of Cd in seawater has long been recognized to have a strong correlation to nutrients, especially phosphate, and behaves similarly [56]. Meaning, Cd is depleted in the surface and enriched in deeper water, where organic matter is decomposed. Higher levels of Cd in deep-sea water explain the higher levels offshore, especially in *B. glaciale* inhabiting deeper waters than *M. muelleri*.

In crustaceans, the maximum limit in the EU regulation 1881/2006 of 0.5 mg/kg w.w. only applies to muscle meat from the abdomen, so the here presented concentrations could not directly be compared. However, the measured concentrations in *Pasiphaea* spp. appeared to be high, also compared to the other two species. The here measured concentrations in *M. norvegica* were low compared to the literature values being one magnitude higher. Cadmium levels in this species seemed to show large variations, also in the literature with large ranges of measurements.

Besides, Antarctic krill *Euphausia superba* from the Western Antarctic Peninsula contains contained higher values of Cd (0.29 mg/kg d.w.), and also other historical measurements have approved this trend [57].

For *E. arcticus*, the literature values from the Mediterranean Sea were comparable to our measurements. However, the comparison to literature values had some flaws. Factors, such as location, season, size, sex, and other physiological factors, might affect trace element content. In addition, the number of measured samples in the different studies was rather low, although mostly pooled samples were analyzed. Furthermore, differences in the used analytical approaches must be considered, especially for work done in the early years. Another factor facilitating differences between studies was the mobility of Cd during sample processing. It has been shown for other crustaceans that freezing and thawing are influencing the distribution of Cd within an animal [58], and as krill decomposes rather fast, a loss of Cd together with other fluids is not unlikely. To get a better understanding of the measured contaminant levels, fatty acid and stable isotope signatures might be analyzed and compared to understand the trophic niche of the different mesopelagic species and how and if the different contaminants are biomagnified in the food-web.

*P. periphylla* had values comparable to the crustaceans and thereby higher values than the fish species.

The JECFA set a provisional tolerable monthly intake (PTMI) for Cd of 25 µg/kg body weight per month, and the European Food Safety Authority (EFSA) announced an even lower tolerable weekly intake for Cd of 2.5 µg/kg body weight [59]. However, even considering the highest concentrations found in the here analyzed mesopelagic species, only unreasonably high consumption would cause health issues.

#### 3.1.3. Mercury

The Hg levels in the fish species *B. glaciale* and *M. muelleri* were low compared to the maximum level of 0.5 mg/kg w.w. and most other commercially used fish species from the North-East Atlantic [60]. Measured concentrations were comparable to the literature values, except two measurements, one from the North Atlantic Ocean with 0.038 mg/kg w.w., and one exceptionally high measurement from the Mediterranean Sea clearly stood out. However, the reason for this could not be explained by the authors either, although local pollution could not be ruled out [31].

While our concentrations measured in the crustaceans *M. norvegica* and *E. arcticus* were considerably lower than the literature values, the concentrations in *Pasiphae* sp. were comparable with the literature values.

The JECFA revised the PTWI for methylmercury (MeHg) in 2007 and reduced it to 1.6 µg/kg body weight per week, and EFSA had set a lower TWI of 1.3 µg/kg body weight per week [61]. Even when assuming a high proportion of MeHg in the measured total Hg, the here measured levels in the mesopelagic organisms were low and not of food safety concern.

#### 3.1.4. Lead

For the two measured fish species, Pb concentrations were low, also in the literature, and far below the EU maximum level in the muscle meat of fish and in whole fish, where fish are intended to be eaten the whole of 0.3 mg/kg w.w.

Moreover, the crustaceans were below the EU maximum level for muscle meat from appendages and abdomen in crustaceans of 0.5 mg/kg w.w., although whole individuals were analyzed. As there is evidence that Pb accumulates heavily in the hepatopancreas of marine shrimps [62,63,64], it can be assumed that the muscle meat of our investigated crustaceans also was below the EU maximum level. Recently, no TWI is in place for Pb, since EFSA in 2010 [65] and JECFA in 2011 [66] withdrew it. It was no longer considered to be protective as there is no evidence for a threshold for critical effects.

Compared to the literature values, our measured concentrations were rather low. However, the literature values for *M. norvegica* did vary much with mean concentrations between <0.3 and 4.65 mg/kg w.w.

#### 3.1.5. Fluoride

Concentrations of fluoride measured in *M. norvegica* were high and comparable to the literature values and concentrations found in Antarctic krill *E. superba*. The concentrations in the other analyzed crustacean species and the jellyfish were much lower.

No maximum level for fluoride is given for foodstuffs in the EU; however, EFSA established a tolerable upper intake level (UL) of fluoride in different age classes [67]. Considering this UL, only low amounts of krill could be consumed, ranging from 2 to 10 g/day depending on the age (Table 3).

In an existing exposure assessment on fluoride, two-year-old children and adults were estimated to exceed the UL, considering exposure from toothpaste, recommended use of dental tablets, the 95-percentile fluoride exposure from drinking water and of tea, and an estimated fixed value of 0.2 mg/day for other exposures [68]. Consequently, it cannot be recommended to consume considerable amounts of krill because of its high content of fluoride.

European Commission set a maximum level for fluorine in feed ingredients with a dry matter of 88% at 500 mg/kg. The only exception was krill, where the upper limit was set to 3000 mg/kg (88% dry matter). However, the final diet concentration must still be below 350 mg/kg (88% dry matter). It was found that Atlantic salmon (*Salmo salar*) was highly tolerant to dietary fluoride given as krill meal with a concentration of fluoride up to 350 mg/kg diet, and that accumulation of fluoride from feeding diets containing krill meal did not lead to tissue accumulation in the fish, at least over a short period of time [69]. Fluorine uptake from krill (*Thysanoessa inermis* and *E. superba*) and the amphipod *Themisto libelulla* was evaluated in Atlantic salmon, Atlantic cod (*Gadus morhua*), rainbow trout (*Onchorhyncus mykiss*), and Atlantic halibut (*Hippoglossus hippoglossus*). Results showed no increase in fluorine levels of any organs and no effects in growth or health [70].

Most of the fluoride in Antarctic krill *E. superba* was found in carapace [10,11] and might be removed, which, however, is tedious, given the small size of krill. It has also been shown that fluoride leaks into the muscle meat of krill post mortem [71]. The fluoride content in krill was shown to be dependent on the molting stage of the krill [10] with much lower values right after ecdysis. In theory, this might be an opportunity to target freshly molted individuals if simultaneous molting takes place.

There is evidence that bioavailability of fluoride from Antarctic krill in mice is high [72] and that it can induce histopathology in livers, kidneys, and bones [73]. However, the actual bioavailability needs to be investigated further for *M. norvegica*. A high bioavailability paired with a high fluoride concentration may make exploitation of *M. norvegica* for direct consumption as food problematic.

#### 3.1.6. Influence of Size, Location, and Sex on Trace Element Concentrations

For Hg in the fish species, we saw a clear indication of higher values of Hg at larger sizes. In *B. glaciale*, we compared three size classes (<40 mm, 45–55 mm, >60 mm) from the same fjord (Osterfjorden) and obtained Hg concentrations of 0.013, 0.025, and 0.044 mg/kg w.w., respectively. For *M. muelleri*, fish above and below 30 mm were compared, and the concentrations were 0.011 and 0.031 mg/kg w.w., respectively. As Hg is known to accumulate over time, this is not unexpected. A similar trend was found earlier in *B. glaciale* [9] and in other North-East Atlantic fish species [60].

For the crustaceans, only the samples of *M. norvegica* allowed a comparison of within the same fjord with individuals below and above 30 mm, and with concentrations of 0.013 and 0.024 mg/kg w.w., there was a clear indication for a size dependency as well.

For Cd, there were differences between the different size classes in both fish species, with higher concentrations in the smallest size classes (*B. glaciale*: <40 mm: 0.044 mg/kg w.w.; 45–55 mm: 0.008 mg/kg w.w., and *M. muelleri*: <30 mm: 0.041; >30 mm: 0.027 mg/kg w.w.). However, in *B. glaciale*, the largest fish (>60 mm) again had higher values than the medium-sized fish with 0.015 mg/kg w.w. No trends could be identified in *M. norvegica*, likely due to the limited amount of samples, as a clear negative correlation with size has been found earlier [35].

For the other trace elements, no clear trends could be identified. However, due to the low number of samples, further research would be desirable investigating the correlation between size and element concentrations, as there might be a potential for targeted harvesting of certain size classes to obtain lower concentrations of undesirable elements.

For *B. glaciale*, males and females were analyzed separately for the medium size class from Osterfjorden, and no trends could be found for any of the analyzed elements, and neither was there a visible trend in elements concentrations between the different fjords in any of the analyzed species.

### 3.2. Dioxins, Furans, PCBs, and Polybrominated Flame-retardants

The sum values of PCBs, dioxins, and furans, summed dioxins and furans (PCDD/F), summed dioxin-like PCBs (Sum dl-PCBs), the sum of these (PCDDF/F + dl-PCBs), the sum of six (PCB_6_) and seven (PCB_7_) indicator PCBs, respectively, and the sum of seven PBDEs (PBDE_7_) in the analyzed samples are given in Table 4. The maximum levels are defined in terms of upper bound sum-parameters [14,74]. The sum-parameters regarding dioxins were measured in the TEQ pg/g w.w. scale (toxic equivalents), in effect, summing toxicities rather than their analytical concentrations, as specified in the regulation (EC) 1881/2006 [14].

None of the measured values exceeded the maximum level for certain contaminants in foodstuffs given in EC1881/2006 [14]. Regarding PCDDF/F + dl-PCBs, it appeared that in all species, except the jellyfish *P. periphylla*, half of the burden was PCDD/F and the other half dl-PCBs.

Literature data on the here measured persistent organic pollutants is scarce for our analyzed species underlining the need for more data. For the fish species, only two studies could be identified for reporting values for reference. One study reported values for *B. glaciale* from a Norwegian fjord and the Northern Norwegian sea, and the values were comparable to our measured concentrations with a mean of 0.51 and 0.59 ng 2005-TEQ/kg w.w. sum PCDD/F, respectively [9]. However, the sum dl-PCBs was higher in our samples with a mean of 0.84 ng 2005-TEQ/kg w.w. compared to means of 0.51 in a Norwegian fjord and 0.42 ng 2005-TEQ/kg w.w. in the Northern Norwegian Sea, also after considering the dry matter content, which was comparable, and higher fat content. This also resulted in a higher value of PCDD/F + dl-PCBs. The mean values for the fish and krill species for the sum PCDD/F + dl-PCB in the present study, ranging from 0.55 to 2.0 ng 2005-TEQ/kg w.w., were similar, although with a higher range, compared to another pelagic species, the Norwegian spring-spawning (NSS) herring, with mean values per sampling position ranging between 0.45 and 1.2 1998-TEQ/kg w.w. [75].

Furthermore, the PCB_6_ content in *B. glaciale* in our measured samples was much higher with a mean of 13 μg/kg w.w., compared to 5.0 and 2.7 μg/kg w.w. in a Norwegian Fjord and the Northern Norwegian Sea, respectively. The same pattern could be seen in PBDE_7_ in *B. glaciale* with a mean of 0.97 μg/kg w.w. compared to 0.24 and 0.46 μg/kg w.w. in a Norwegian Fjord and the Northern Norwegian Sea, respectively. This indicated local differences, which also got evident when comparing our data from the three different fjords. Osterfjorden showed much higher concentrations of sum dl-PCBs, sum PCB_6/7_, and PBDE_7_ compared to Boknafjorden and Bjørnafjorden with rather similar values (Supplementary Table S1). The same trend was found for all other species, except the jellyfish, and thereby indicated a higher level of pollution due to a local source for these substances in Osterfjorden. The second set of literature values suggested low values in the North Atlantic with concentrations of 0.22 and 0.350 ng WHO 2005-TEQ/kg of PCDD/F and PCDDF/F + dl-PCBs [30]. Large differences in concentrations were also found in a study investigating the accumulation of dioxins in deep-sea crustaceans in the Mediterranean. In *Pasiphaea multidentata*, they measured 0.90 ng WHO 1998-TEQ/kg of PCDD/F inside a submarine canyon, while outside 1.5 ng WHO 1998-TEQ/kg was found. Both values were rather high compared to our measured concentration of 0.44 ng WHO 1998-TEQ/kg in *Pasiphaea* spp. [76].

In *B. pterotum*, fished in the Gulf of Oman, the concentrations of PCDD/F and PCDD/F + dl-PCBs were measured to be 0.1 and 0.249 TEQ ng/kg d.w., respectively [8]; however, it is not given which TEQ was used. Our values measured in *B. glaciale* of 2.5 and 4.95 TEQ ng/kg dry weight, respectively, were much higher, and further investigations are needed to understand species differences in this closely related species.

Considering the different length classes from the same fjord analyzed in the two fish species, there was a clear indication of a positive relationship between the here measured persistent organic pollutants and size, similar to what was found for other organic pollutants in freshwater fish species [77]. We also observed a trend towards higher concentrations in females of *B. glaciale* having the same fat content as males, which could be caused by sexual growth dimorphism, with females of certain species investing more energy in reproduction, and thereby growing slower and having more time to accumulate persistent contaminants than males at the same size. However, sexual growth dimorphism was not found in this species in the Northwestern Atlantic [78] or at the Flemish cape [79], and neither the mercury concentrations were different between males and females, and further investigations are needed.

### 3.3. Lipid Compounds

The lipid contents are presented in Table 5, and fatty acid and fatty alcohol profiles are given in Supplementary Table S2. The fish species were the most lipid-rich of the studied organisms with 18 ± 8% fat in *M. muelleri* and 14 ± 4% fat in *B. Glaciale.* Followed by the shrimps with 9 ± 3% fat *in E. Arcticus* and 5 ± 3% fat in *Pasiphae* spp. Northern krill, *M. Norvegica* had 5 ± 1% fat, while the jellyfish—*P. periphylla—*was very lean, containing only 0.5 ± 0.2% fat.

#### 3.3.1. Wax Esters

Two of our investigated species—*B. glaciale* and *E. arcticus—*were storing energy as wax esters (long-chain fatty alcohols esterified to long-chain fatty acids), and the wax esters contributed with 64% and 46% to the total lipid, respectively. In *M. muelleri, M. norvegica*, and *Pasiphaea* spp., only traces of wax esters were detected, constituting 0.2–1.5% of the total lipid. These originated most likely from calanoid specimens present in the stomach and digestion system. In *P. periphylla*, the wax esters contributed 22% to the total lipid; however, as the level of lipid was only 0.45% of the wet weight, it is also likely that these wax esters originated from *Calanus* prey.

Wax esters are common lipid in many mesopelagic invertebrates and fish, where it functions both as energy reserves and buoyancy regulator [80,81].

Since wax esters are not properly absorbed in the mammalian digestive tract [82], and a high intake can lead to oily diarrhea, also called keriorrhea [83], they do pose a food safety concern [84,85]. Keriorrhea has mainly been observed after the consumption of the two fish species—oilfish (*Ruvettus pretiosus)* and escolar (*Lepdocybium flavobrunneum).* Both have a high-fat content of about 20%, of which 90% are wax esters, resulting in a wax ester content in the fillet of up to 18% [85,86]. There have not been conducted any clinical studies on the effects of consumption of oilfish, but from volunteer testing, it has been reported that an intake of 140 g of escolar (corresponding to 25 g wax esters) could induce keriorrhea [85]. A portion of about 300 g whole *B. glaciale* would deliver a comparable amount of wax esters, so keriorrhea might be a problem if consuming a large amount of this fish. To our knowledge, no tolerable intake of wax esters has been established by any authority so far, and further studies are needed to get a better understanding of keriorrhea and if it can be induced by the consumption of mesopelagic fish-containing wax esters.

The safety of human consumption of wax ester rich oil from copepods (*Calanus finmarchicus*) was studied through clinical trials (randomized, double-blind, placebo-controlled), and long-term consumption (12 months) of 2 g/day did not show any negative effects on hematological and clinical chemistry parameters, including gastrointestinal-related effects [87,88]. Despite a large amount of wax ester, *calanus* oil has been suggested as a good source of the long-chain polyunsaturated fatty acids (PUFA), eicosapentaenoic acid, and docosahexaenoic acid for humans, and commercial products are available as dietary supplements [89,90].

The use of calanus oil as lipid sources for salmon feed has also been intensively studied [91]. Salmon has a limited ability to digest wax esters, and these lipids should not exceed 30% of the dietary lipid, so the high amount of wax ester in some of the mesopelagic species has to be taken into account when used for fish feed [84,92,93]. However, the mesopelagic species are also a good source of (n-3) PUFAs and can, therefore, be a valuable marine-based lipid source for fish feed [7,47].

#### 3.3.2. Erucic Acid

Erucic acid is naturally present in the marine food chain, and the EFSA published a risk assessment of erucic acid (22:1n-9) in 2016, where a tolerable daily intake (TDI) for humans of 7 mg/kg body weight per day was established [94]. The concern about erucic acid in the human diet is focused on the consumption of plant oils, like mustard oil, in which erucic acid can make up to 50% of the total FAs. An ML for erucic acid in fish and seafood has not yet been considered [95]. Long-chain monounsaturated fatty acids (≥20 carbon) are poorly catabolized through normal β-oxidation in the mitochondria and induce instead peroxisomal β-oxidation, where the FAs are shortened into C18 MUFAs, which then can be further catabolized in mitochondrial β-oxidation [96]. Diets with high levels of erucic acid have been associated with myocardial lipidosis and cardiovascular diseases [94]. In marine sources, the (n-11) isomer is often dominating the (n-9)-isomers, and in the present mesopelagic marine species, the amount of cetoleic acid(22:1 (n-11) was 7–25 times higher than erucic acid. In contrast to the health concerns indicated for erucic acid, cetoleic acid has been reported to stimulate the synthesis of eicosapentaenoic acid and docosahexaenoic acid from α-linolenic acid in human HepG2 and of EPA in salmon hepatocytes in vitro and to increase whole-body retention of EPA + DHA in salmon [97].

*M. muelleri* had the highest levels of 22:1 acids, and consumption of around 400 g of this fish would give levels of erucic acid (480 mg) exceeding the EFSA TDI in a 70 kg person. However, further studies regarding its metabolism and its health effects in fish and humans are needed to improve risk-benefit assessments since there also is evidence that the consumption of oily fish not necessarily is associated with negative effects on cardiovascular health [95,98].

### 3.4. Undesirables in Processed Mesopelagic Biomass

#### 3.4.1. Estimates for Fish Meal and Protein Fraction

The results of our estimates of the concentration of undesirables in fish meal, assuming that they completely follow the protein fraction, are given in Table 6.

Assuming that the whole biomass will be processed to fish meal with 10% fat content, a meal consisting of *Pasiphae* sp. only would exceed the ML in fish feed for Hg, while a mixed catch without jellyfish would exceed the maximum level in fish feed for fluorine. If only the protein fraction is considered to be used for fish feed production, the maximum level for Cd would be exceeded in *Pasiphaea* spp. and *P. periphylla.* The As, Hg, and fluoride ML would be exceeded in all measured species. In addition, if the determination of iAs is required by competent authorities (EU Directive 2002/32 and amendments [15]), the iAs concentration would be exceeded in *P. periphylla* and in mixed catches with jellyfish. The Hg and fluoride maximum level would be exceeded in all measured species. Interestingly, due to its large contribution in the actual catches, the jellyfish *P. periphylla* is responsible for very high Cd values in the protein fraction only estimate, exceeding the maximum level 10-fold. However, as we do not have enough knowledge on the actual processing factors and if the protein fraction will be used in feed or for human consumption, the here estimated values have high uncertainty. We are aware that it is not likely that processed fish meal only will consist of the protein fraction, and some elements might even be eliminated during the processing or follow the oil fraction as described for Cd in marine oils from calanus [99]. The final complete fish feed will be composed of different ingredients, and the fish meal from the here investigated species will only contribute to a minor part of the feed composition. The results of the applied crude protein are also somewhat uncertain as we assumed a standard amino acid composition and that all measured nitrogen originates from protein. Future studies should take into account the amino acid profile to be able to calculate the true protein content [100,101]. The here calculated numbers are results of a worst-case scenario and were only used to identify possible issues.

#### 3.4.2. Estimates for Fish Oil

The estimates of dioxins, furans, and PCBs in fish oil after processing, assuming that all these will end up completely in the oil fraction, are given in Table 7.

Oil produced from all species would exceed the maximum levels given in the EU Directive 2002/32/EC on undesirable substances in animal feed for PCDD/F (EU, 2002) and the Commission Regulation (EC) No 1881/2006 setting maximum levels for certain contaminants in foodstuffs [14]. The same would apply for oil produced from a bulk average catch with and without contribution from jellyfish in the investigated fjords (see Alvheim et al. [7] for catch composition). *P. periphylla* surprisingly showed the highest concentration of PCDD/F, probably due to its low-fat content. For PCDD/F + dl-PCB and PCB_6_, none of the produced oils from the species nor the average catches would exceed the maximum level in animal feed. However, all would be above the maximum level in marine oil intended for human consumption. Nevertheless, many of the currently sold marine oils are cleaned before being sold, which may also be feasible for oils originating from mesopelagic species and grant them marked access.

Samples for this investigation have been taken in December, and there is evidence that the fat content also in mesopelagic species is varying with season [47], which, in turn, might influence the load of persistent organic pollutants and should, therefore, be investigated further.

### 3.5. General Discussion

In our study, we analyzed the contaminants in mesopelagic biomass on the species level. Catches of mesopelagic species have been shown to vary significantly in terms of species composition [30,47]. Our data showed a large variation in a load of undesirables of the species, and the contaminant load of a catch would vary accordingly. Therefore, our species-specific data is of high value as it can be used to predict the contaminant profile of a catch if the species composition of the catch is known. The commercial mesopelagic fishery is still under development, and for the moment, it is impossible to predict the main use of this resource. A targeted fishery for some more valuable species and/or fishing for bulk biomass for processing can be imagined. Regardless of the outcome, species-specific data on undesirables will be of significant value. As the genus or family of the here investigated species are found widespread and highly abundant in mesopelagic ecosystems all around the globe, our data allows predictions for other species and possible fisheries as well.

However, further investigations are needed to get a better understanding of the dynamics of contaminants, including nutrients in mesopelagic species, from different regions to assess spatial variation. Regarding harvesting the mesopelagic species, seasonal differences should especially be assessed to facilitate a targeted harvest of the most suitable biomass for food and feed. As feeding patterns vary throughout the year [102], differences in the body, species, and size composition of the catches are likely.

Our results showed that mesopelagic species from Western Norwegian fjords might be suitable for direct consumption with the exception of *M. norvegica* due to the high fluoride values. *B. glaciale* might have some limitations regarding the levels of wax esters and *M. muelleri* regarding erucic acid.

Considering our predictions of undesirables in the fish meal fraction, in the protein fraction, and oil fraction after processing, we were able to identify possible food and feed safety issues. Regarding fish meal, the predictions are less accurate, as we do not know enough about the actual processing. However, total As might be a challenge, but a better understanding of the processing, bioavailability, and speciation is needed before conclusions can be drawn. In the protein fraction, several undesirable elements showed concentrations above the MLs if the protein fraction was intended directly for human consumption. In virgin marine oils made of the here investigated mesopelagic species, the content of PCDD/F would be likely to exceed the maximum level for non-human consumption (fish oil) and especially human consumption, and also the content of PCDD/F + dl-PCB would be too high for commercial trade for human consumption of marine oils. However, refining and cleaning methods are also applied in other marine oils to remove organic pollutant contamination. To be able to make more precise predictions, processing factors for the different relevant product scenarios should be established in future studies. In addition, other contaminants should be taken into consideration, like chlorinated pesticides or microplastics. As observed for other marine organisms (reviewed in Kögel et al. [103]), microplastic has also been reported in the digestive tract of mesopelagic fish species (*B. glaciale, M. muelleri*, and *Notoscopelus kroyeri*) in 11% of the individuals [104].

The knowledge created in the present study is crucial to enable an evaluation of the value of these species. The ecological role these animals might play in terms of carbon pumping is not fully understood yet, but there are clear indications that mesopelagic organisms are having a direct influence on the global CO_2_ budget and thereby climate change [13,105]. Large-scale harvesting of mesopelagic biomass should, therefore, be postponed until we know what we actually can win or lose by harvesting the different species and applying different processing methods.

## Figures and Tables

**Table 1:** Concentrations of total arsenic (As), inorganic arsenic (iAs), cadmium (Cd), mercury (Hg), lead (Pb), fluoride (F) (Mean ± SD) on wet weight (w.w.) and dry weight (d.w.) basis in the most abundant mesopelagic species in western Norwegian fjords. An asterisk indicates upper bound mean concentrations.

**Species**	**N**	**As**		**iAs**		
		**(g/kg w.w.)**	**(mg/kg d.w.)**	**(mg/kg w.w.)**		**(mg/kg d.w.)**
		**Mean ± SD**		***N***	**Mean ± SD**	
*Benthosema glaciale*	7	4.0 ± 1.2 (2.2–6.0)	13 ± 4 (6.9–19)	3	<LOQ	<LOQ
*Maurolicus muelleri*	4	5.1 ± 0.5 (4.7–5.5)	16 ± 1 (15–17)	3	<LOQ	<LOQ
*Meganyctiphanes norvegica*	4	28 ± 19 (12–52)	89 ± 61 (38–160)	3	0.061 ± 0.086 (0.011–0.160)	0.244 ± 0.348 (0.042–0.646)
*Pasiphaea* sp.	3	22 ± 19 (10–43)	68 ± 58 (32–136)	3	0.014 ± 0.010 (0.007–0.025)	0.061 ± 0.032 (0.042–0.098)
*Eusergestes arcticus*	4	9.5 ± 4.2 (5.0–14)	30 ± 13 (16–44)	3	<LOQ	<LOQ
*Periphylla periphylla*	2	0.79 (0.59–1.0)	2.5 (1.9–3.2)	2	0.0022 (0.0021–0.0023)	0.046 (0.044–0.048)
**Species**	**N**	**Cd**		**Hg**		
		**(mg/kg w.w.)**	**(mg/kg d.w.)**	**(mg/kg w.w.)**		**(mg/kg d.w.)**
		**Mean ± SD**				
*Benthosema glaciale*	7	0.022 ± 0.014 (0.007–0.044)	0.069 ± 0.043 (0.022–0.14)	0.022 ± 0.012 (0.011–0.044)		0.069 ± 0.037 (0.035–0.14)
*Maurolicus muelleri*	4	0.033 ± 0.007 (0.026–0.041)	0.1 ± 0.02 (0.082–0.13)	0.026 ± 0.011 (0 011–0.035)		0.080 ± 0.033 (0.035–0.11)
*Meganyctiphanes norvegica*	4	0.016 ± 0.013 0.008–0.035)	0.051 ± 0.04 (0.025–0.11)	0.014 ± 0.007 (0 008–0.024)		0.044 ± 0.022 (0.025–0.076)
*Pasiphaea* sp.	3	0.26 ± 0.19 (0.14–0.47)	0.81 ± 0.58 (0.44–1.5)	0.038 ± 0.02 (0.022–0.060)		0.12 ± 0.06 (0.069–0.19)
*Eusergestes arcticus*	4	0.074 ± 0.042 (0.029–0.13)	0.23 ± 0.13 (0.092–0.41)	0.014 ± 0.007 (0.008–0.023)		0.043 ± 0.021 (0.025–0.073)
*Periphylla periphylla*	2	0.075 (0.064–0.085)	0.24 (0.20–0.27)	<LOQ		<LOQ
**Species**	**N**	**Pb**		**F**		
		**(mg/kg w.w.)**	**(mg/kg d.w.)**	**(mg/kg w.w.)**		**(mg/kg d.w.)**
		**Mean ± SD**				
*Benthosema glaciale*	7	0.016 ± 0.017 * (<LOQ–0.054)	0.049 ± 0.054 * (<LOQ–0.17)	-		-
*Maurolicus muelleri*	4	0.009 ± 0.001 * (<LOQ–0.010)	0.027 ± 0.004 * (<LOQ–0.032)	-		-
*Meganyctiphanes norvegica*	4	0.086 ± 0.075 (0.021–0.16)	0.27 ± 0.24 (0.066–0.51)	720 ± 160 (570–940)		3000 ± 500 (2700–3700)
*Pasiphaea* spp.	3	0.005 ± 0.002 * (<LOQ–0.006)	0.016 ± 0.005 * (<LOQ–0.019)	63 ± 8 (57–72)		300 ± 60 (240–360)
*Eusergestes arcticus*	4	0.01 ± 0.006 * (<LOQ–0.019)	0.032 ± 0.019 * (<LOQ–0.060)	27 ± 11 (18–42)		100 ± 60 (60–190)
*Periphylla periphylla*	2	<LOQ	<LOQ	8		168

* Upper bound concentration.

**Table 2:** Literature values of measured trace element concentrations (As, Cd, Hg, MeHg, Pb, and F) in the investigated species. The number of measured samples (N), the mean concentration in mg/kg based on either dry weight (d.w.) or wet weight (w.w.), the standard deviation (SD), and the range, whenever available, are given.

Species	Element	Location	N	Mean (mg/kg)	SD	Range	d.w./w.w.	Reference	
*Benthosema glaciale*	As	North Atlantic	1 ^c^	0.58			w.w.	[30]	Grimaldo et al. in press
		N Norwegian Sea	25 ^c^	1.4		1.2–1.8	w.w.	[9]	Wiech et al., 2018
		Norwegian Coast	4 ^c^	1.9		1.8–2.0	w.w.	[9]	Wiech et al., 2018
		Mediterranean Sea	1	12.7			d.w.	[31]	Fowler, 1986
	Cd	North Atlantic	1 ^c^	0.090			w.w.	[30]	Grimaldo et al. in press
		N Norwegian Sea	25 ^c^	0.067		0.044–0.086	w.w.	[9]	Wiech et al., 2018
		Norwegian Coast	4 ^c^	0.009		0.006–0.018	w.w.	[9]	Wiech et al., 2018
		Mediterranean Sea	9 ^c^	0.71	0.15		d.w.	[31]	Fowler, 1986
		Mediterranean Sea	4 ^c^	0.19	0.08		d.w.	[31]	Fowler, 1986
	Hg	North Atlantic	1 ^c^	0.039			w.w.	[30]	Grimaldo et al. in press
		N Norwegian Sea	25 ^c^	0.019		0.014–0.024	w.w.	[9]	Wiech et al., 2018
		Norwegian Coast	4 ^c^	0.016		0.013–0.020	w.w.	[9]	Wiech et al., 2018
		Mediterranean Sea	9 ^c^	0.4	0.16		d.w.	[31]	Fowler, 1986
		Mediterranean Sea	11 ^c^	0.21	0.2		d.w.	[31]	Fowler, 1986
	Pb	North Atlantic	1 ^c^	<0.01			w.w.	[30]	Grimaldo et al., in press
		N Norwegian Sea	25 ^c^	0.021		0.007–0.089	w.w.	[9]	Wiech et al., 2018
		Norwegian Coast	4 ^c^	0.008		0.007–0.010	w.w.	[9]	Wiech et al., 2018
*Maurolicus muelleri*	As	North Atlantic	2 ^c^	1.6		1.2–1.9	w.w.	[30]	Grimaldo et al. in press
		Norwegian Fjord	4 ^c^	3.8		2.5–4.6	w.w.	[9]	Wiech et al., 2018
	Cd	North Atlantic	2 ^c^	0.38		0.31–0.44	w.w.	[30]	Grimaldo et al., in press
		Norwegian Fjord	4 ^c^	0.026		0.018–0.032	w.w.	[9]	Wiech et al., 2018
	Hg	North Atlantic	2 ^c^	0.026		0.022–0.030	w.w.	[30]	Grimaldo et al., in press
		Norwegian Fjord	4 ^c^	0.034		0.024–0.049	w.w.	[9]	Wiech et al., 2018
		Azores	11	0.34		0.051–0.446	d.w.	[32]	Monteiro et al., 1996
	Pb	North Atlantic	2 ^c^	<0.05			w.w.	[30]	Grimaldo et al., in press
		Norwegian Fjord	4 ^c^	0.009		0.006–0.014	w.w.	[9]	Wiech et al., 2018
*Meganyctiphanes norvegica*	As	NE Atlantic	5	59.3	11.0		d.w.	[33]	Ridout et al., 1989
		NE Atlantic	8	42			d.w.	[34]	Leatherland et al., 1973
		Mediterranean Sea	1 ^c^	55.8			d.w.	[31]	Fowler, 1986
	Cd	NE Atlantic	29	0.66		0.14–1.83	w.w.	[35]	P. S. Rainbow, 1989
		NE Atlantic	5	0.39	0.03		d.w.	[33]	Ridout et al., 1989
		NE Atlantic	29	1.6	1.2		d.w.	[33]	Ridout et al., 1989
		NE Atlantic	8	0.25			d.w.	[34]	Leatherland et al., 1973
		North Sea/Atlantic	18 ^c^	0.54	0.10		d.w.	[36]	Zauke et al., 1996
		Greenland Sea	19 ^c^	0.44	0.10		d.w.	[37]	Ritterhoff and Zauke, 1997
		Atlantic/Firth of Clyde	30	1.06		0.54–6.06	w.w.	[35]	P. S. Rainbow, 1989
		Mediterranean Sea	5 ^c^	1.3			d.w.	[31]	Fowler, 1986
		Mediterranean Sea	2 ^c^	0.12			d.w.	[38]	Fossi et al., 2002
		Mediterranean Sea	1	1.06			d.w.	[39]	Belloni et al., 1976
		Mediterranean Sea/Corsica	4	0.55	0.03		d.w.	[40]	Roméo and Nicolas, 1986
		Mediterranean Sea/Monaco	n.a.	0.74			d.w.	[41]	Fowler, 1977
		NE Pacific	9	2.8		0.8–5.5	d.w.	[42]	Martin and Knauer, 1973
	Hg	NE Atlantic	8	0.26			d.w.	[34]	Leatherland et al., 1973
		Mediterranean Sea	2 ^c^	0.14			d.w.	[38]	Fossi et al., 2002
		Mediterranean Sea/Monaco	n.a.	0.35			d.w.	[31]	Fowler, 1986
		Mediterranean Sea	1	0.092			d.w.	[31]	Fowler, 1986
		Gulf of St Lawrence	6 ^c^	0.60	0.05		d.w.	[43]	Lavoie et al., 2010
	Mehg	Gulf of St Lawrence	5 ^c^	0.065	0.03		d.w.	[43]	Lavoie et al., 2010
	Pb	Greenland Sea	17 ^c^	<0.3			d.w.	[37]	Ritterhoff and Zauke, 1997
		Mediterranean Sea/Corsica	4	4.65	2.11		d.w.	[40]	Roméo and Nicolas, 1986
		Mediterranean Sea/Monaco	n.a.	1.1			d.w.	[41]	Fowler, 1977
		Mediterranean Sea	2 ^c^	0.50			d.w.	[38]	Fossi et al., 2002
		NE Pacific	9	2.4		1.0–10.9	d.w.	[42]	Martin and Knauer, 1973
	F-	W-Sweden/N-Kattegat	6	2153			d.w.	[10]	Adelung et al., 1987
		Norwegian Coast	2 ^c^	1845		1330–2360	d.w. ^f^	[11]	Soevik and Braekkan, 1979
*Pasiphaea* spp.	Hg	Gulf of St Lawrence	2 ^c^	0.11	0.02		d.w.	[43]	Lavoie et al., 2010
		Gulf of Maine	8 ^c^	0.27	0.07	0.166–0.347	w.w.	[44]	Harding et al., 2018
	Mehg	Gulf of Maine	8 ^c^	0.15	0.11	0.03–0.351	w.w.	[44]	Harding et al., 2018
*Eusergestes arcticus*	Cd	Mediterranean Sea	6 ^c^	0.90		0.4–1.5	d.w.	[31]	Fowler, 1986
		Mediterranean Sea/Corsica	5	0.33	0.17	0.12–0.52	d.w.	[40]	Roméo and Nicolas, 1986
	Hg	Mediterranean Sea	1	0.31			d.w.	[31]	Fowler, 1986
	Pb	Mediterranean Sea/Corsica	4	2.13	0.36	1.71–2.38	d.w.	[40]	Roméo and Nicolas, 1986

^c^ composite samples; ^f^ fat-free dry weight; n.a.: not available.

**Table 3:** Amount of the most common mesopelagic crustaceans from Norwegian fjords in grams that can be consumed before exceeding the daily tolerable upper intake level of fluoride (UL) proposed by the European food safety authority * in different age classes of consumers.

Age (*y*)	UL (mg/day)	*M. norvegica* (g)	*Pasiphaea* spp. (g)	*E. arcticus* (g)
1 to 3	1.5	2.1	24	56
4 to 8	2.5	3.5	40	93
9 to 14	5	6.9	81	185
≥15	7	9.7	113	259

* Opinion of the Scientific Panel on Dietetic Products, Nutrition and Allergies on a request from the Commission related to the Tolerable Upper Intake Level of Fluoride [67].

**Table 4:** The sum values of PCBs, dioxins, furans, and polybrominated flame-retardants furans in the most abundant mesopelagic species in Western Norwegian fjords. Summed dioxins and furans (PCDD/F), summed dioxin-like PCBs (Sum dl-PCBs), the sum of these (PCDDF/F + dl-PCBs), the sum of six (PCB_6_) and seven (PCB_7_) indicator PCBs, respectively, and the sum of seven PBDEs (PBDE_7_) (Mean ± SD; Min-Max) on wet weight basis are given. Maximum levels for certain contaminants in foodstuffs given in regulation EC1881/2006 are shown for comparison.

Species	N	Sum PCDD/F	Sum dl-PCBs	PCDD/F + dl-PCBs	PCB_6_	PCB_7_	PBDE_7_
		(ng 2005-TEQ/kg w.w.)			(μg/kg w.w.)		
		Mean ± SD / (Min–Max)					
*B. glaciale*	5	0.77 ± 0.21 (0.46–1.03)	0.84 ± 0.44 (0.53–1.6)	1.6 ± 0.6 (1.1–2.6)	13 ± 11 (3.5–26)	15 ± 12 (4.1–31)	0.97 ± 0.68 (0.40–1.8)
*M. muelleri*	4	1.1 ± 0.6 (0.43–1.8)	0.97 ± 0.54 (0.42–1.6)	2.0 ± 1.0 (0.85–3.0)	13 ± 8 (5.4–25)	15 ± 10 (6.2–29)	1.0 ± 0.4 (0.63–1.5)
*M. norvegica*	3	0.29 ± 0.06 (0.23–0.35)	0.26 ± 0.17 (0.15–0.45)	0.54 ± 0.22 (0.38–0.79	5.8 ± 6.3 (1.9–13)	6.7 ± 7.2 (2.1–15)	0.42 ± 0.26 (0.25–0.72)
*Pasiphaea* spp.	3	0.37 ± 0.16 (0.22–0.55)	0.28 ± 0.13 (0.13–0.36)	0.66 ± 0.28 (0.35–0.90)	5.5 ± 5.6 (1.4–12)	6.3 ± 6.4 (1.6–14)	0.45 ± 0.27 (0.19–0.72)
*E. arcticus*	4	0.83 ± 0.32 (0.54–1.27	0.72 ± 0.35 (0.41–1.1)	1.6 ± 0.6 (0.94–2.2)	10 ± 8 (3.4–21)	12 ± 9 (3.9–24)	0.75 ± 0.39 (0.39–1.3)
*P. periphylla*	2	0.064 (0.038–0.089)	0.011 (0.011–0.012)	0.075 (0.048–0.10)	0.049 (0.042–0.056)	0.053 (0.046–0.061)	0.010 (0.008–0.011)
Maximum level		3.5	-	6.5	75	-	-

**Table 5:** The content of fatty acids, fatty alcohols, wax esters, long-chain monounsaturated fatty acids (erucic acid, cetoleic acid), and total fat (Mean ± SD; Min–Max) in the most abundant mesopelagic species in Western Norwegian fjords.

Species	Fatty Acids	Fatty Alcohols	Wax Esters	Erucic Acid 22:1 (n-9)	Cetoleic Acid 22:1 (n-11)	Total Fat Content
	(µg/100 µg w.w.)		% of fatty acids	(µg/100 µg w.w.)		
	Mean ± SD / (Min–Max)					
*Benthosema glaciale*	6.8 ± 1.8 (3.1–7.8)	4.2 ± 1.2 (1.8–5.1)	76	0.05 ± 0.02 (0.02–0.07)	0.78 ± 0.24 (0.26–1.07)	13.7 ± 3.7 (6.1–16.0)
*Maurolicus muelleri*	14.5 ± 7.9 (5.3–21.1)	0.03 ± 0.01 (0.02–0.05)	<0.5	0.12 ± 0.08 (0.03–0.20)	3.1 ± 1.8 (0.7–4.6)	17.8 ± 8.1 (7.1–24.7)
*Meganyctiphanes norvegica*	4.2 ± 0.8 (3.3–4.9)	0.07 ± 0.02 (0.06–0.09)	<1.5	0.03 ± 0.02 (0.002–0.05)	0.26 ± 0.22 (0.012–0.54)	5.5 ± 0.6 (4.9–5.9)
*Pasiphaea* spp.	3.7 ± 1.8 (2.4–5.7)	0.02 ± 0.01 (0.01–0.03)	<0.5	0.03 ± 0.02 (0.013–0.05)	0.20 ± 0.15 (0.19–0.29)	5.4 ± 2.7 (3.3–8.4)
*Eusergestes arcticus*	5.3 ± 2.1 (2.6–7.8)	2.4 ± 1.0 (1.1–3.3)	46	0.04 ± 0.02 (0.01–0.05)	0.52 ± 0.23 (0.01–0.05)	9.4 ± 3.1 (4.9–12.1)
*Periphylla periphylla*	0.19 (0.15–0.22)	0.04 (0.01–0.08)	22	0.003 ± 0.001 (0.001–0.003)	0.027 ± 0.011 (0.011–0.035)	0.45 (0.34–0.56)

**Table 6:** Estimated concentrations of trace elements (total arsenic (As), inorganic arsenic (iAs), cadmium (Cd), mercury (Hg), lead (Pb), fluoride (F)), dioxins, furans, dioxin-like polychlorinated biphenyls, polybrominated flame-retardants, erucic acid, and wax esters in processed mesopelagic biomass with a dry matter content of 88%, assuming that the respective trace elements will end up completely in protein/fish meal after processing. (A) shows the estimates for a fish meal with 10% fat content and (B) for the protein fraction only. Maximum levels given in EU Directive 2002/32/EC on undesirable substances in animal feed are given for comparison.

	**(A) Fish Meal with 10% Fat and 88% Dry Matter**								**(B) Protein Fraction with 88% Dry Matter**						
	**As**	**iAs**	**Cd**	**Hg**	**Pb**	**F**			**As**	**iAs**	**Cd**	**Hg**	**Pb**	**F**	
**Species**		**[mg/kg w.w.]**													
*B. glaciale*	12	0	0.065	0.065	0.046	0			82	0	0.45	0.44	0.003	0	
*M. muelleri*	15	0	0.094	0.074	0.025	0			110	0	0.75	0.55	0.002	0	
*M. norvegica*	99	0.21	0.057	0.049	0.30	2500			670	1.4	0.30	0.33	0.020	13000	
*Pasiphaea* spp.	84	0.054	0.99	0.15	0.019	240			620	0.40	5.2	1.1	0.001	1200	
*E. arcticus*	30	0	0.24	0.044	0.033	84			200	0	1.4	0.28	0.002	630	
*P. periphylla*	13	0.036	1.2	0.033	0.17	132			1500	4.2	22	3.8	0.19	3400	
Average catch ^1^ wo jellyfish	38	0.052	0.13	0.062	0.099	610 ^2^			260	0.35	0.76	0.43	0.007	3200 ^2^	
Average catch ^1^ w jellyfish	14	0.051	1.2	0.034	0.16	155 ^2^			1500	4.0	21	3.7	0.18	3400 ^2^	
Maximum level ^3^	25	-	2	0.1	10	3000 ^4^		500 ^5^	25	-	2	0.1	10	3000 ^4^	500 ^5^
	**Sum PCDD/F**	**Sum dl-PCB**	**PCDD/F + dl-PCB**	**PCB_6_**	**PCB_7_**	**PBDE_7_**	**Eurucic acid**	**Wax esters**							
	**[ng 2005-TEQ/kg w.w.]**			**[μg/kg w.w.]**			**[µg/100 µg w.w.]**								
*B. glaciale*	0.56	0.61	1.2	9.5	1.1	7.1	0.04	3.8							
*M. muelleri*	0.62	0.54	1.1	7.3	8.4	5.6	0.07	0.04 ^6^							
*M. norvegica*	0.53	0.47	0.98	11	1.2	7.6	0.06	0.03 ^6^							
*Pasiphaea* spp.	0.69	0.52	1.2	10	12	8.3	0.06	0.03 ^6^							
*E. arcticus*	0.88	0.77	1.7	11	13	8.0	0.04	2.6							
*P. periphylla*	0.14	0.20	1.7	1.1	1.2	2.2	0.07	0.93							
Average catch ^1^ wo jellyfish	0.61	0.59	1.2	9.6	11	7.2	0.05	2.0 ^6^							
Average catch ^1^ w jellyfish	1.4	0.26	1.6	1.5	1.7	2.5	0.07	9.8 ^6^							
Maximum level ^3^	5.0	-	20	175	-	-									

^1^ Average catch composition is shown in Alvheim et al. [7]; ^2^ Assuming the two fish species containing no fluoride at all; ^3^ Given in the EU Directive 2002/32/EC on undesirable substances in an5imal feed [15]; ^4^ Maximum level only applies to marine crustaceans, such as marine krill; ^5^ Maximum level applies to feed materials of animal origin except marine crustaceans; ^6^ Upper bound values.

**Table 7:** Estimated mean concentrations of summed dioxins and furans (PCDD/F), summed dioxin-like PCBs (Sum dl-PCBs), the sum of these (PCDDF/F + dl-PCBs), the sum of six (PCB_6_) and seven (PCB_7_) indicator PCBs, the sum of seven PBDEs (PBDE_7_), erucic acid, and wax esters, assuming that the respective undesirables will end up completely in fish oil after processing. Maximum levels for non-human consumption (NHC) and human consumption (HC) given in EU Directive 2002/32/EC and Commission Regulation (EC) No 1881/2006, respectively, are given for comparison.

Species	N	Sum PCDD/F	Sum dl-PCBs	PCDD/F + dl-PCB	PCB_6_	PCB_7_	PBDE_7_	Erucic Acid	Wax Esters
		(ng 2005-TEQ/kg w.w.)			(μg/kg w.w.)			(µg/100 µg w.w.)	
*Benthosema glaciale*	5	5.6	6.1	12	95	110	7.1	0.36	38
*Maurolicus muelleri*	4	6.2	5.4	11	73	84	5.6	0.67	0.41 ^4^
*Meganyctiphanes norvegica*	3	5.3	4.7	9.8	110	120	7.6	0.55	0.27 ^4^
*Pasiphaea* spp.	3	6.9	5.2	12	100	120	8.3	0.56	0.34 ^4^
*Eusergestes arcticus*	4	8.8	7.7	17	110	130	8.0	0.43	26
*Periphylla periphylla*	2	14	2.0	17	11	12	2.2	0.67	9.3
Average catch ^1^ wo jellyfish		6.1	5.9	12	96	110	7.2	0.47	20 ^4^
Average catch ^1^ w jellyfish		14	2.6	16	15	17	2.5	0.66	9.8 ^4^
Maximum level ^2^	NHC ^2^	5.0	-	20	175	-	-		-
	HC ^3^	1.75	-	6.0	200	-	-		-

^1^ Average catch composition is shown in Alvheim et al. [7]; ^2^ Given in the EU Directive 2002/32/EC on undesirable substances in animal feed [15]; ^3^ Given in the EU Regulation 1881/2006 setting maximum levels for certain contaminants in foodstuffs [14]; ^4^ Upper-bound estimates.

## Supplementary Materials

The following are available online at https://www.mdpi.com/2304-8158/9/9/1162/s1, Table S1: Concentrations of trace elements, dioxins, furans, PCBs, polybrominated flame-retardants, erucic acid, and wax esters in the most abundant mesopelagic species in Western Norwegian fjords on individual sample level., Table S2: Fatty acids and fatty alcohol profiles in the most abundant mesopelagic species in Western Norwegian fjords on the individual sample level.
