# Supplementary material for: Undesirables in Mesopelagic Species and Implications for Food and Feed Safety—Insights from Norwegian Fjords
Source: Foods. 2020 Aug 24;9(9):1162. doi: 10.3390/foods9091162 (PMC7555207; doi:10.3390/foods9091162)
Supplement: Supplementary file 1 [file foods-09-01162-s001.pdf]

Supplementary Material to "Undesirables in mesopelagic species and implications for food and feed safety - Insights from Norwegian fjords"

Table S1. Concentrations of trace elements, dioxins, furans, polychlorinated biphenyls and polybrominated flame-retardants in the most abundant mesopelagic species in Western Norwegian Fjords on individual composite sample level.

Species	Area	N per sample	Size / weight	Length class	Sex	Dry matter	Total fat	As	Cd	Hg	Pb	Fluoride	PCDD/F	dl-PCBs	PCDD/F + dl-PCBs	PCB_6	PCB_7	$PBDE_7$
			[mm] / [g]	[mm]		[%]	[%]	[mg/kg w.w.]					[ng 2005-TEQ/kg w.w.			[µg/kg w.w.]		
Benthosema glaciale	Osterfjorden	27	63 ± 2	>60	17m 11f	32	15	4.7	0.015	0.044	0.009	-	-	-	-	-	-	-
		135	50 ± 3	45-55	m	32	16	3.6	0.007	0.021	0.01	-	0.79	0.89	1.7	22	26	1.6
		135	49 ± 3	45-55	f	32	15	3.7	0.008	0.028	0.011	-	1.0	1.6	2.6	26	31	1.8
		>50	24 ± 6	<40	immature	22	6.1	6	0.044	0.013	0.011	-	0.46	0.65	1.1	9.9	12	0.64
		15	50 ± 3	-	-	31		3.7	0.027	0.022	0.054	-	-	-	-	-	-	-
	Boknafjorden	>50	53 ± 6	-	-	32	15	2.2	0.034	0.011	< .007	-	0.80	0.56	1.4	3.8	4.4	0.4
	Bjørnafjorden	83	41 ± 11	-	-	34	16	4.1	0.018	0.013	< .007	-	0.78	0.53	1.3	3.5	4.1	0.41
Maurolicus muelleri	Osterfjorden	>50	24 ± 3	<30	-	23		5.5	0.041	0.011	0.01	-	0.43	0.42	0.85	11	13	0.63
		>50	45 ± 4	>30	-	31	16	5.5	0.027	0.031	< .007	-	1.2	1.6	2.8	25	29	1.5
	Boknafjorden	>50	48 ± 6	-	-	41	23	4.7	0.036	0.035	< .009	-	1.8	1.3	3.0	10	12	1.2
	Bjørnafjorden	>50	37 ± 9	-	-	38	25	4.7	0.026	0.025	< .008	-	0.81	0.61	1.4	5.4	6.2	0.68
Meganyctiphanes norvegica	Osterfjorden	>50	17 ± 2	<30	-	21		52	0.008	0.013	0.14	570	-	-	-	-	-	-
		>50	33 ± 2	>30	-	25	4.9	36	0.009	0.024	0.021	940	0.35	0.45	0.79	13	15	0.72
	Boknafjorden	>50	33 ± 2	-	-	25	5.9	12	0.035	0.011	0.021	660	0.29	0.17	0.45	2.6	2.9	0.29
	Bjørnafjorden	>50	30 ± 5	-	-	25	5.8	13	0.013	0.008	0.16	710	0.23	0.15	0.38	1.9	2.1	0.25
Pasiphaea spp.	Osterfjorden	>50	70 ± 11	-	-	24	3.3	43	0.14	0.060	< .006	72	0.35	0.36	0.71	12	14	0.72
	Boknafjorden	>50	82 ± 8	-	-	16	8.4	10	0.47	0.033	0.003	57	0.55	0.35	0.90	3.3	3.7	0.43
	Bjørnafjorden	>50	49 ± 20	-	-	25	4.6	12	0.16	0.022	< .006	60	0.22	0.13	0.35	1.4	1.6	0.19
Eusergestes arcticus	Osterfjorden	>50	32 ± 5	-	-	22	4.9	14	0.029	0.008	0.006	42	0.54	0.41	0.94	11	12	0.64
		>50	26 ± 5	-	-	31	12.1	12	0.075	0.023	< .007	18	0.87	1.1	2.0	21	24	1.3
	Boknafjorden	>50	50 ± 10	-	-	28	10.6	5	0.13	0.01	0.009	23	0.65	0.43	1.1	3.4	3.9	0.39
	Bjørnafjorden	>50	44 ± 7	-	-	29	10	6.9	0.063	0.014	0.019	23	1.3	0.95	2.2	5.6	6.4	0.67
Periphylla periphylla	Osterfjorden	12	575 ± 446	-	-	5	0.34	1	0.085	< .002	< .01	8	0.038	0.011	0.048	0.056	0.061	0.011
	Bjørnafjorden	10	952 ± 292	-	-	5	0.56	0.59	0.064	< .002	< .01		0.089	0.012	0.10	0.042	0.046	0.0083

Table S2. Fatty acids and fatty alchols profiles in the most abundant mesopelagic species in Western Norwegian Fjords on individual composite sample level.

	Benthosema glaciale (n=8)	Maurolicus muelleri (n=4)	Meganyctiphanes norvegica (n=4)	*Pasiphaea sp.* (n=3)	Eusergestes arcticus (n=4)	*Periphyalla periphylla* (n=2)
**Amount FA (µg/100 µg sample weight))**	**6.8 ± 1.7**	**14.5 ± 7.9**	**3.4 ± 1.7**	**3.7 ± 1.8**	**5.3 ± 2.1**	**0.19 ± 0.04**
**Amount FAOH (µg/100 µg sample weight))**	**4.3 ± 1.1**	**0.0 ± 0.0**	**0.05 ± 0.03**	**0.0 ± 0.0**	**2.4 ± 1.0**	**0.04 ± 0.05**
**FA profile (% of total FAs)**						
14:0	5.00 ± 0.52	7.05 ± 0.60	5.07 ± 1.03	2.42 ± 0.78	3.34 ± 0.75	3.03 ± 0.97
15:0	0.22 ± 0.02	0.52 ± 0.10	0.71 ± 0.09	0.37 ± 0.07	0.27 ± 0.05	0.28 ± 0.05
16:0	5.82 ± 0.51	16.14 ± 2.01	15.22 ± 0.41	15.92 ± 0.09	9.00 ± 0.78	9.64 ± 1.51
Iso 17:0	0.25 ± 0.02	0.24 ± 0.06	0.36 ± 0.06	0.36 ± 0.04	0.21 ± 0.02	0.29 ± 0.04
17:0	0.18 ± 0.04	0.28 ± 0.10	0.38 ± 0.13	0.36 ± 0.08	0.22 ± 0.05	0.35 ± 0.02
18:0	1.45 ± 0.29	1.66 ± 0.44	2.18 ± 0.27	2.80 ± 0.09	1.01 ± 0.20	4.43 ± 0.71
**∑SFA**	**13.04 ± 1.48**	**26.52 ± 2.40**	**24.73 ± 1.08**	**23.02 ± 0.84**	**14.49 ± 1.76**	**18.48 ± 1.83**
16:1 (n-9)	0.34 ± 0.03	0.20 ± 0.04	0.31 ± 0.09	0.25 ± 0.04	0.26 ± 0.02	0.12 ± 0.02
16:1 (n-7)	11.23 ± 0.87	5.55 ± 0.21	4.52 ± 0.38	3.66 ± 0.25	7.59 ± 0.29	3.45 ± 0.14
16:1 (n-5)	0.27 ± 0.05	0.27 ± 0.05	0.26 ± 0.06	0.16 ± 0.05	0.41 ± 0.09	0.15 ± 0.13
17:1 (n-8)	0.25 ± 0.24	0.31 ± 0.08	0.36 ± 0.07	0.43 ± 0.06	0.38 ± 0.05	0.36 ± 0.09
18:1 (n-11)	0.50 ± 0.13	0.34 ± 0.09	0.15 ± 0.08	0.18 ± 0.04	3.35 ± 2.21	0.41 ± 0.16
18:1 (n-9)	19.84 ± 3.13	9.40 ± 1.78	13.37 ± 3.45	21.76 ± 3.54	15.12 ± 4.03	16.57 ± 7.34
18:1 (n-7)	1.95 ± 0.15	2.01 ± 0.15	4.83 ± 0.65	5.79 ± 0.44	3.40 ± 0.72	3.13 ± 0.64
18:1 (n-5)	0.40 ± 0.04	0.35 ± 0.04	0.34 ± 0.04	0.41 ± 0.04	0.63 ± 0.08	0.67 ± 0.04
20:1 (n-11)	1.90 ± 0.28	1.02 ± 0.16	0.71 ± 0.19	0.97 ± 0.18	2.99 ± 0.80	1.44 ± 0.47
20:1 (n-9)	7.63 ± 1.08	9.99 ± 2.23	5.90 ± 3.22	4.71 ± 1.16	9.93 ± 2.32	9.70 ± 0.31
20:1 (n-7)	0.36 ± 0.05	0.22 ± 0.02	0.63 ± 0.11	0.45 ± 0.11	0.47 ± 0.11	3.22 ± 0.21
22:1 (n-11)	11.31 ± 1.45	20.40 ± 4.83	6.13 ± 4.05	4.91 ± 1.50	9.47 ± 2.10	14.39 ± 2.63
22:1 (n-9)	0.66 ± 0.11	0.81 ± 0.15	0.81 ± 0.42	0.73 ± 0.20	0.72 ± 0.16	1.42 ± 0.22
22:1 (n-7)	0.26 ± 0.03	0.24 ± 0.02	0.22 ± 0.04	0.32 ± 0.01	0.22 ± 0.02	0.31 ± 0.03
24:1 (n-9)	1.41 ± 0.27	1.27 ± 0.26	0.64 ± 0.06	0.85 ± 0.05	0.69 ± 0.29	0.63 ± 0.02
**∑MUFA**	**57.08 ± 5.83**	**52.72 ± 5.82**	**39.63 ± 6.57**	**46.10 ± 4.07**	**56.03 ± 4.05**	**56.67 ± 5.68**
16:2 (n-4)	0.85 ± 0.09	0.64 ± 0.09	0.46 ± 0.15	0.21 ± 0.10	0.52 ± 0.07	0.25 ± 0.02
18:2 (n-6)	1.81 ± 0.16	1.32 ± 0.09	2.75 ± 1.09	1.70 ± 0.43	2.19 ± 0.39	1.22 ± 0.02
20:2 (n-6)	0.24 ± 0.02	0.18 ± 0.02	0.33 ± 0.03	0.39 ± 0.02	0.37 ± 0.02	0.36 ± 0.00
20:4 (n-6)	0.56 ± 0.08	0.35 ± 0.10	1.24 ± 0.73	1.12 ± 0.42	0.70 ± 0.21	0.96 ± 0.11
22:4 (n-6)	0.09 ± 0.01	0.04 ± 0.01	0.09 ± 0.00	0.05 ± 0.00	0.06 ± 0.04	0.37 ± 0.05
22:5 (n-6)	0.17 ± 0.02	0.14 ± 0.04	0.40 ± 0.15	0.28 ± 0.07	0.19 ± 0.05	0.07 ± 0.00
18:3 (n-3)	1.42 ± 0.13	1.05 ± 0.18	1.26 ± 0.24	0.86 ± 0.27	1.52 ± 0.19	0.81 ± 0.13
18:4 (n-3)	2.26 ± 0.43	1.76 ± 0.32	1.55 ± 0.74	0.99 ± 0.62	1.63 ± 0.16	1.05 ± 0.26
20:3 (n-3)	0.15 ± 0.01	0.14 ± 0.03	0.39 ± 0.05	0.40 ± 0.08	0.26 ± 0.02	0.48 ± 0.03
20:4 (n-3)	0.98 ± 0.10	0.47 ± 0.11	0.47 ± 0.13	0.42 ± 0.09	1.03 ± 0.07	0.87 ± 0.20
20:5 (n-3)	6.17 ± 0.59	4.30 ± 0.76	10.23 ± 2.67	10.34 ± 1.11	8.85 ± 0.84	6.60 ± 1.60
22:4 (n-3)	0.08 ± 0.01	0.09 ± 0.03	0.04 ± 0.03	0.05 ± 0.01	0.10 ± 0.01	0.33 ± 0.04
22:5 (n-3)	0.90 ± 0.07	0.73 ± 0.07	0.81 ± 0.08	0.61 ± 0.06	0.99 ± 0.08	6.76 ± 1.72
22:6 (n-3)	10.39 ± 1.60	8.19 ± 2.45	14.19 ± 3.06	12.10 ± 1.18	9.05 ± 1.18	2.64 ± 0.26
24:5 (n-3)	0.40 ± 0.07	0.52 ± 0.09	0.32 ± 0.09	0.24 ± 0.01	0.50 ± 0.02	0.68 ± 0.15
20:2 NMI	0.17 ± 0.03	0.08 ± 0.03	0.23 ± 0.08	0.35 ± 0.07	0.19 ± 0.07	0.27 ± 0.15
**∑PUFA**	**27.14 ± 3.32**	**20.76 ± 3.65**	**35.64 ± 7.14**	**30.88 ± 3.56**	**29.48 ± 2.49**	**24.85 ± 3.85**
**∑PUFA (n-6)**	**3.23 ± 0.27**	**2.25 ± 0.23**	**5.03 ± 1.85**	**3.69 ± 0.79**	**3.80 ± 0.69**	**3.33 ± 0.32**
**∑PUFA (n-3)**	**22.51 ± 3.08**	**17.53 ± 3.47**	**29.69 ± 5.67**	**26.36 ± 2.95**	**24.32 ± 1.90**	**20.47 ± 4.30**
**FAOH profile (% of total FAOHs)**						
14:0 Alk	4.33 ± 0.74	3.19 ± 6.38	2.23 ± 1.56	4.07 ± 1.13	4.82 ± 0.23	2.74 ± 0.23
15:0 Alk	0.72 ± 0.07	0.00 ± 0.00	0.35 ± 0.26	0.17 ± 0.29	0.42 ± 0.07	0.39 ± 0.07
16:0 Alk	25.95 ± 1.74	14.62 ± 4.37	15.41 ± 7.10	14.44 ± 1.83	20.25 ± 5.65	24.42 ± 5.65
18:0 Alk	2.60 ± 0.45	21.92 ± 7.90	5.97 ± 6.93	3.55 ± 1.30	1.18 ± 0.24	1.76 ± 0.24
20:0 Alk	0.31 ± 0.03	3.38 ± 3.43	2.48 ± 3.46	2.06 ± 1.02	0.23 ± 0.11	0.50 ± 0.11
24:0 Alk	0.28 ± 0.03	2.73 ± 3.91	3.64 ± 3.77	2.97 ± 2.00	0.75 ± 0.10	0.65 ± 0.10
16:1 (n-7) Alk	3.11 ± 0.14	0.41 ± 0.82	1.96 ± 1.32	1.30 ± 0.99	5.08 ± 0.15	2.97 ± 0.15
18:1 (n-9) Alk	11.85 ± 2.56	3.37 ± 3.01	4.91 ± 4.45	4.29 ± 1.29	4.14 ± 1.58	2.48 ± 1.58
18:1 (n-7) Alk	3.32 ± 0.46	0.22 ± 0.43	1.62 ± 1.21	1.74 ± 0.30	1.99 ± 0.50	1.72 ± 0.50
18:1 (n-5) Alk	0.66 ± 0.02	2.57 ± 3.97	0.27 ± 0.26	6.53 ± 2.97	0.48 ± 0.01	0.41 ± 0.01
∑20:1 Alk (dominated by (n-9))	15.02 ± 1.68	20.95 ± 2.63	25.23 ± 10.72	24.78 ± 3.45	17.61 ± 2.21	19.85 ± 2.21
∑22:1 Alk (dominated by (n-11))	29.71 ± 3.29	23.04 ± 12.35	34.19 ± 11.59	31.98 ± 5.16	41.29 ± 5.82	39.87 ± 5.82
18:2 (n-6) Alk	1.01 ± 0.15	1.01 ± 1.22	0.60 ± 0.40	0.88 ± 0.22	0.87 ± 0.03	0.57 ± 0.03

Fatty acids that contribute to less than 0.3 % of the total FA profiles are not included in the table, but are still part of the sum of total FAs. This include iso 15:0, 20:0, 21:0, 22:0, 24:0, 14:1 (n-5), 16:1 (n-10)-7 me, 16:1 (n-11), 20:1 (n-5), 24:1 (n-7), 16:4 (n-1), 18:4 (n-1), 16:3 (n-4), 18:2 (n-4), 16:2 (n-6), 18:3 (n-6), 20:3 (n-6), 22:2 (n-6), 16:4 (n-3). These minor FAs contribute together with less than 2 % of the total FAs.
